# Novel Methodology for the Design of Personalized Cancer Vaccine Targeting Neoantigens: Application to Pancreatic Ductal Adenocarcinoma

**DOI:** 10.3390/diseases12070149

**Published:** 2024-07-11

**Authors:** Kush Savsani, Sivanesan Dakshanamurthy

**Affiliations:** 1 Department of Surgery, Virginia Commonwealth University, Richmond, VA 23219, USA; 2 Lombardi Comprehensive Cancer Center, Georgetown University School of Medicine, Washington, DC 20007, USA

**Keywords:** personalized cancer vaccines, neoantigens, pancreatic ductal adenocarcinoma, peptide based personalized cancer vaccine, MHC, HLA, TCR

## Abstract

Personalized cancer vaccines have emerged as a promising avenue for cancer treatment or prevention strategies. This approach targets the specific genetic alterations in individual patient’s tumors, offering a more personalized and effective treatment option. Previous studies have shown that generalized peptide vaccines targeting a limited scope of gene mutations were ineffective, emphasizing the need for personalized approaches. While studies have explored personalized mRNA vaccines, personalized peptide vaccines have not yet been studied in this context. Pancreatic ductal adenocarcinoma (PDAC) remains challenging in oncology, necessitating innovative therapeutic strategies. In this study, we developed a personalized peptide vaccine design methodology, employing RNA sequencing (RNAseq) to identify prevalent gene mutations underlying PDAC development in a patient solid tumor tissue. We performed RNAseq analysis for trimming adapters, read alignment, and somatic variant calling. We also developed a Python program called SCGeneID, which validates the alignment of the RNAseq analysis. The Python program is freely available to download. Using chromosome number and locus data, SCGeneID identifies the target gene along the UCSC hg38 reference set. Based on the gene mutation data, we developed a personalized PDAC cancer vaccine that targeted 100 highly prevalent gene mutations in two patients. We predicted peptide-MHC binding affinity, immunogenicity, antigenicity, allergenicity, and toxicity for each epitope. Then, we selected the top 50 and 100 epitopes based on our previously published vaccine design methodology. Finally, we generated pMHC-TCR 3D molecular model complex structures, which are freely available to download. The designed personalized cancer vaccine contains epitopes commonly found in PDAC solid tumor tissue. Our personalized vaccine was composed of neoantigens, allowing for a more precise and targeted immune response against cancer cells. Additionally, we identified mutated genes, which were also found in the reference study, where we obtained the sequencing data, thus validating our vaccine design methodology. This is the first study designing a personalized peptide cancer vaccine targeting neoantigens using human patient data to identify gene mutations associated with the specific tumor of interest.

## 1. Introduction

Personalized cancer vaccines are a rising innovation in the field of vaccine design [1]. These vaccines induce an antigen-specific CD8^+^ and CD4^+^ T-cell response to enhance anti-tumor activity based on a patient’s individual tumor. Technological innovation has led to the ability to rapidly sequence and analyze patient genome data, which led to the selection of gene targets and on-demand production of personalized therapy [2]. A phase I clinical trial synthesized personalized mRNA vaccines against PDAC from solid tumors, which led to improved disease-free survival [3]. The trial analyzed a patient population who underwent surgical resection of PDAC tumors. Future development of personalized cancer vaccines directly to demonstrate significant efficacy in patients without major surgical intervention.

Pancreatic ductal adenocarcinoma (PDAC) is the most common form of pancreatic cancer and is projected to be the second-leading cause of cancer mortality by 2030 [4,5]. Current clinical therapies involve neoadjuvant therapy followed by possible surgical resection [6]. However, patients with PDAC suffer from poor prognosis, with a median survival rate of 22.1 months and an actual survival rate of 17.0% [7]. PDAC is often diagnosed late, and as a result, surgical resection may not be a viable option for many patients [8]. As the cancer progresses and possible treatment options decrease, survival outcomes also significantly worsen. The five-year survival rate for patients diagnosed with late-stage PDAC is less than 10% [8].

PDAC progresses as a complex activation of driver genes and inactivation of tumor suppressor genes [9]. Commonly mutated genes observed in PDAC include KRAS, TP53, CDNK2A, DPC4/SMAD4, and BRCA2. Studies of key mutations in these genes are conducted with the goal of developing targeted gene therapies. One particular mutation, the KRAS G12D mutation, is present in over 40% of PDAC patients [10]. However, this specific mutation has been found not to be significantly associated with overall survival outcomes. The TP53 gene is mutated in about 50% of PDAC patients [11]. These mutations include gain-of-function point mutations and null mutations as a result of deletions. Mutations of the CDNK2A gene have been found to be significantly associated with poorer survival outcomes for patients with PDAC compared to mutations of KRAS and TP53 [12,13].

Several PDAC vaccines are under development, and clinical trials are being conducted using a variety of immunologic targeting methods [14]. These methods include cell-based, protein-based, microorganism-based, DNA-based, exosome-based, and peptide-based vaccines. Peptide-based vaccines have been growing in popularity due to their ability to be quickly and cheaply developed and their flexibility in patient populations [15]. For PDAC, the first peptide vaccine to undergo clinical trials was a KRAS-targeting peptide co-administered with GM-CSF to promote a greater immune response [16]. The vaccine successfully induced specific immune responses in 58% of patients, contributing to a longer survival time for treated patients. Other peptide vaccines targeting survivin, gastrin, VEGFR-1, VEGFR-2, and WT1 have been ineffective in inducing immune response or contributing to significantly improved survival [14,16,17,18,19,20]. However, the design of personalized-based peptide cancer vaccines is completely absent. This study focuses on the development of a design protocol to create personalized peptide vaccines with application to PDAC. The protocol identifies genetic variants using RNAseq analysis and designs a personalized peptide vaccine using a vaccine development protocol and omics pipeline previously developed by our group [21].

## 2. Materials and Methods

### 2.1. Patient Genomic Data

We obtained patient genomic data from the Gene Expression Omnibus (GEO) database [22], a publicly accessible repository of comprehensive microarray, next-generation sequencing, and other forms of high-throughput functional genomic data. For this study, we specifically collected raw Illumina sequencing data pertaining to human patient solid tumor samples. These samples were part of a detailed study focused on analyzing long-term heterogeneity in patients with pancreatic ductal adenocarcinoma (PDAC) [23]. This study included genomic data from a cohort of 19 patients, consisting of 10 long-term and 9 short-term survivors, providing a diverse basis for examining genetic variations linked to survival outcomes. For the objectives of this study, we selected one patient classified as a short-term survivor and one patient classified as a long-term survivor to design personalized vaccines, serving as a proof-of-concept for our approach. This selection was strategic, allowing us to explore the potential of personalized medicine in cases with poorer prognoses and to evaluate the efficacy of targeted therapies based on genomic insights. The design and development of the vaccine were personalized to the unique genetic profile of the chosen patient, focusing on the anomalies most likely to influence tumor behavior and treatment response. To confirm that our personalized vaccine design was rigorous and potentially effective, we compared the targeted genetic components of the vaccine to key genes previously identified as significant in the survival of PDAC patients by Bhardwaj et al. [23]. This comparison enabled us to validate our personalized vaccine design approach and increase the therapeutic relevance of the vaccine design. This properly controlled process of data selection and comparison with established genetic markers supports our vaccine design methodology, which is detailed further in the section below.

### 2.2. RNAseq Analysis of Patient Data

**Figure 1:**
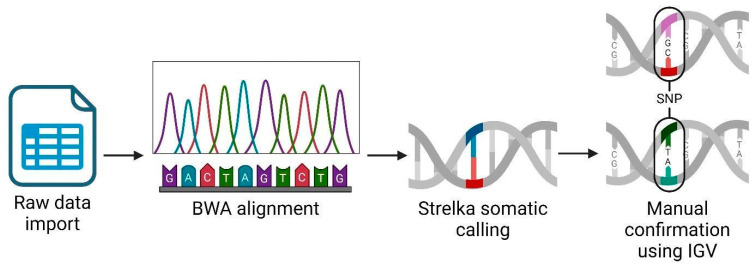
RNAseq analysis workflow using Partek Flow suite. Created using BioRender.com.

We performed an RNAseq analysis using the Partek Flow genomic analysis suite, as shown in Figure 1, which outlines our comprehensive RNAseq workflow to obtain and confirm variant data. Initially, we imported the raw sequence data in a fastq format into Partek Flow. This format is widely used for storing the output from high-throughput sequencing instruments and contains both nucleotide sequence data and corresponding quality scores. Following data importation, the first computational step involved trimming the Illumina sequencing adapters. These adapters, which are artificial sequences added during library preparation, can interfere with the analysis if not removed, as they may be misinterpreted as part of the genomic sequence. After trimming, we aligned the reads to a reference genome using the Burrows–Wheeler Aligner (BWA) algorithm version 0.7.18. BWA is a software tool that efficiently aligns relatively short sequences (such as those from Illumina sequencers) against a long reference sequence, such as a complete genome. This alignment is important for locating the genomic origins of each read and is fundamental to identifying variations from the reference sequence. In the post-alignment, we executed somatic variant calling using the Strelka algorithm, which was specifically designed to detect somatic variants with high sensitivity and accuracy in tumor-normal paired samples. This step was important for identifying potentially significant genetic mutations that could be relevant in the context of disease, herein cancer. To ensure the reliability of our findings, we manually inspected each significant gene variant using the Integrative Genomics Viewer (IGV) (Partek Inc., Chesterfield, Missouri). IGV is an interactive visualization tool that allows us to visually explore genomic data, thus facilitating the validation of computational predictions through a critical human-oversight step. We excluded gene variants of inadequate quality from further analysis. This quality control step is key to avoiding false positives that could skew the results of downstream applications, such as vaccine development. Finally, we focused our efforts on analyzing single nucleotide polymorphisms (SNPs) that held potential for inclusion in our vaccine development process. SNPs, being the most common type of genetic variation among cancer patients, provide valuable insights into genetic variability, which can be exploited to design targeted vaccines.

## 3. Gene Annotation Confirmation Using SCGeneID Python Program

### Development and Application of SCGeneID

**Figure 2:**
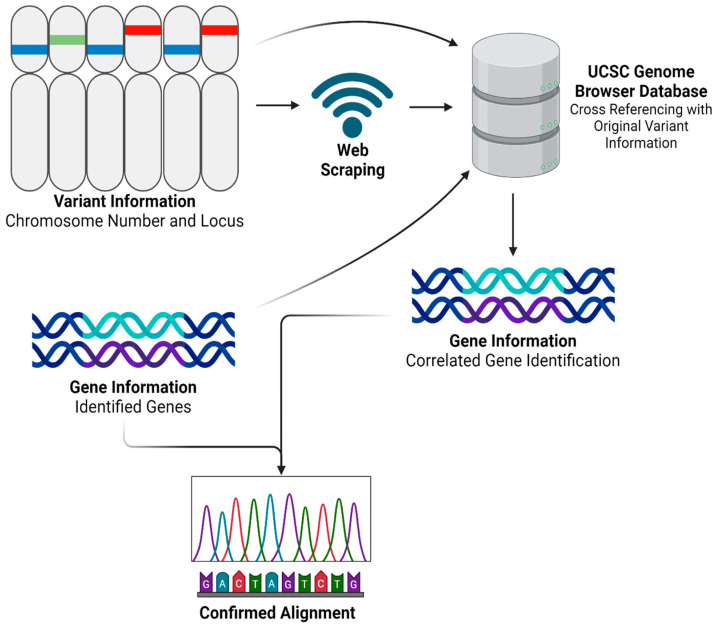
SCGeneID Python program workflow. Created using BioRender.com.

After obtaining and processing genomic data through Partek Flow, we advanced to the next step by developing a Python program named ‘SCGeneID’. The code for this innovative tool is comprehensively detailed in Supplementary File S1 and is freely available for download. SCGeneID was specifically designed to enhance our analytical capabilities in gene annotation by using both chromosome number and locus information. Using the hg38 reference set accessible via the UCSC Genome Browser [24], SCGeneID systematically identifies corresponding gene names based on their chromosomal location. The tool operates by exploiting web-scraping techniques to extract relevant genomic data directly from the browser. Once the data are retrieved, SCGeneID processes this information to generate a detailed output that includes a table formatted with chromosome numbers, locus details, and the names of associated genes. This functionality not only streamlines the gene identification process but also warrants accuracy by referencing updated genomic data. The application of SCGeneID in our study was twofold. Primarily, it served to externally validate the alignment accuracy and overall reliability of our RNAseq analysis process. By cross-verifying the gene annotations provided by Partek Flow with those extracted by SCGeneID, we could confirm the consistency and validity of our results. Additionally, as shown in Figure 2, we employed a modified version of SCGeneID to specifically extract a list of genes from a given variant file. This adaptation was particularly important for our personalized vaccine as it allowed us to focus on particular genomic variants of interest, facilitating a more targeted approach in our subsequent analyses.

## 4. Personalized Vaccine Design Protocol

We employed a vaccine design protocol that has been previously outlined in our published studies [21]. This protocol integrates cutting-edge bioinformatics tools to predict and select epitopes from mutations identified in genomic data.

### 4.1. Epitope Prediction and Selection

Initially, we used the IEDB NetMHC 4.1 tool to predict epitopes. NetMHC 4.1 is specifically designed to return potential epitopes along with their predicted binding affinity for the top 27 expressed HLA alleles in the human population. The binding affinity indicated by the IC_50_ value measured in nanomolar (nM) determines the strength of the interaction between the epitope and the HLA molecules, which is a critical factor in the immune response efficacy.

### 4.2. Clinical Checkpoint Parameters

Subsequently, we computed several epitope-specific clinical checkpoint parameters. The immunogenicity of each epitope was determined using the IEDB Class I Immunogenicity Tool, which assesses the potential of an epitope to trigger an immune response. The antigenicity, which evaluates the capability of the epitope to be recognized by antibodies, was determined using VaxiJen v2.0.

### 4.3. Data Filtering and Selection Criteria

With the binding affinity, immunogenicity, and antigenicity data computed for each epitope and its associated HLA allele, we employed stringent filters to select the most promising epitopes. These filters were applied based on the criteria outlined in Table 1, focusing on identifying epitopes that are strong binders, highly immunogenic, and antigenic.

### 4.4. Physicochemical Property Assessment

In addition to these functional assessments, we analyzed various physicochemical properties of the epitopes using ProtParam https://web.expasy.org/protparam/ (Accessed on 23 September 2023). This analysis included determining parameters such as half-life, instability index, isoelectric point, aliphatic index, and GRAVY score. Although these parameters were informative for understanding the physical and chemical characteristics of the epitopes, they were not used in the epitope selection process. Further, we assessed toxicity using ToxinPred and screened for allergenic potential using AllerTOP v2.0, ensuring that only non-toxic and non-allergenic epitopes were considered for further analysis.

### 4.5. Epitope Selection and Workflow Integration

After applying the filtration restrictions (Table 1), we selected the top 50 and 100 epitopes that met all the specified criteria, warranting a robust selection of candidates for potential vaccine design. We employed binary filters on toxicity and allergenicity to ensure the selection of epitopes that were both non-toxic and non-allergenic.

### 4.6. Methodological Workflow

**Figure 3:**
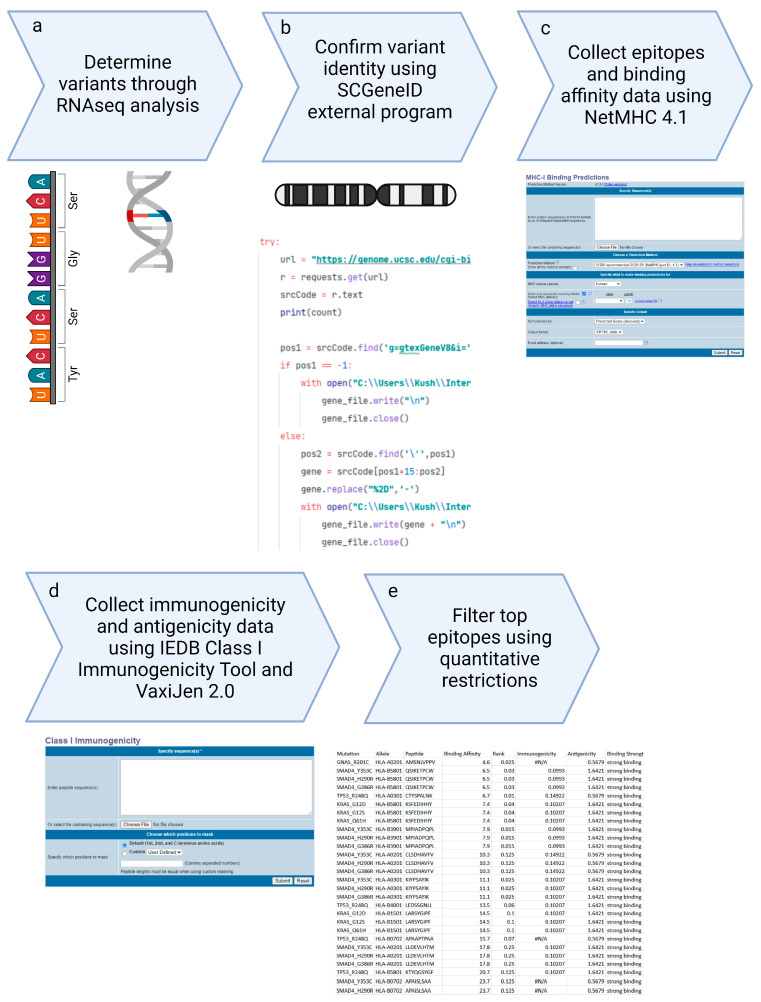
Peptide Based Personalized Cancer Vaccine Design methodological overall workflow.

Figure 3 shows the comprehensive workflow of our methodology, starting from the collection of Illumina sequencing data, performing RNAseq analysis, and the selection of top epitopes for the development of peptide vaccines. This streamlined workflow integrates multiple stages of data processing and epitope evaluation, indicating the robustness of our approach in vaccine design.

## 5. Results

We obtained Illumina sequencing data from two patients out of the 19 available in the GEO accession project [23]. The sequencing data represent the genetic landscape of the patient’s solid tumor sample. We performed RNAseq analysis to determine prevalent mutations. Using these mutations, we determined strong and normal binding MHC class I epitopes that are immunogenic, antigenic, non-toxic, and non-allergenic. We selected the top 50 and top 100 epitopes from these data for a peptide vaccine.

### 5.1. Determination of Genetic Variants with RNAseq Analysis

We performed RNAseq analysis on Illumina sequencing data to obtain a list of genetic variants identified in a solid PDAC tumor. For Patient 1, the RNAseq analysis performed using Partek Flow resulted in 100,819 mutations. These mutations included single-nucleotide polymorphisms, multi-nucleotide polymorphisms, deletions, and insertions. Isolating the single-nucleotide polymorphisms, we identified 189 unique variants, which we could use to develop the peptide vaccine. For Patient 2, the RNAseq analysis resulted in 87,128 mutations., of which we identified 125 unique variants we could use to develop the peptide vaccine.

### 5.2. Confirmation of Genetic Variants and Sequencing Alignment Using SCGeneID

We confirmed the alignment of the sequencing data to the hg38 human reference genome using our SCGeneID program version 1. Using SCGeneID, we qualitatively identified the corresponding genes to all mutations obtained through RNAseq analysis loci against the hg38 human reference genome. We found 100% similarity between the genes identified through Partek Flow and genes identified using SCGeneID for both Patients 1 and 2. Therefore, we were confident that the variant genes identified using Partek Flow were correctly aligned to the reference genome.

### 5.3. Collection of 9-Mer and 10-Mer Top Epitopes from Genetic Variants

From the pool of identified genetic variants, we curated lists of the top 50 and top 100 epitopes, prioritized based on their binding affinity and immunogenic properties. The top epitopes for Patients 1 and 2 can be found in Supplementary Files S2–S5. All selected epitopes consisted of 9 or 10 amino acids, representing an epitope capable of binding to an MHC class I molecule. All the top 50 epitopes were classified as having strong binding affinity to their associated HLA allele. The top 100 epitopes included both strong and normal binders. We found no epitopes in the top 100, which were classified as weak binders. Table 2 shows the top 50 epitopes for Patient 1, along with their associated genes, mutations, and binding HLA alleles.

### 5.4. Population Coverage Analysis of Top 100 Epitopes

We also performed a population coverage analysis to assess the extent of the global population that could potentially benefit from the personalized vaccine. The analysis for Patient 1 showed that the vaccine could cover 69.64% of the global population. Table 3 provides this coverage along with average hit rates and PC_90_ data for various world subregions. While the population coverage may appear relatively low at first glance, it is essential to consider the context of this study. The vaccine was uniquely designed based on the gene expression profile of a specific individual, making it personalized and tailored to the specific mutations and characteristics of their tumor. Consequently, the expectation for widespread coverage across diverse populations is not high. As the patient cohort from whom the vaccine was developed predominantly comprised individuals with European ancestry, the vaccine’s performance in these regional subgroups aligned with the genetic background of the patients involved.

### 5.5. 3D-Structure Modeling of Epitope-MHC and TCR Interaction Complex

**Figure 4:**
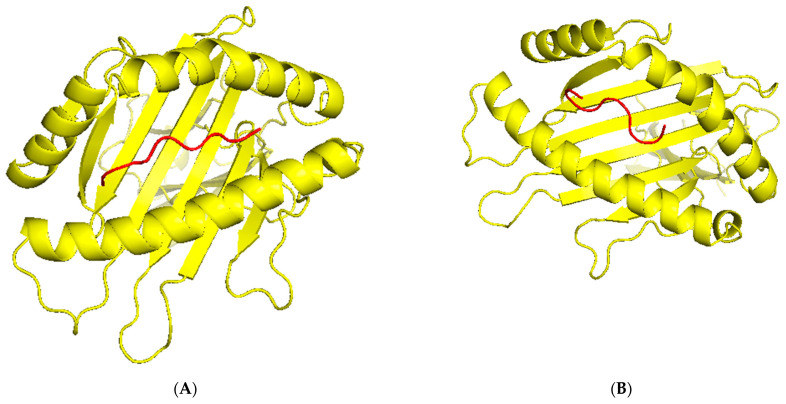
(**A**) The peptide KSFEDIHHY, a mutation of the KRAS gene, bound to MHC Class I molecule HLA-B*58:01. (**B**) The peptide KTYQGSYGF, a mutation of the TP53 gene, bound to MHC Class I molecule HLA-B*58:01.

**Figure 5:**
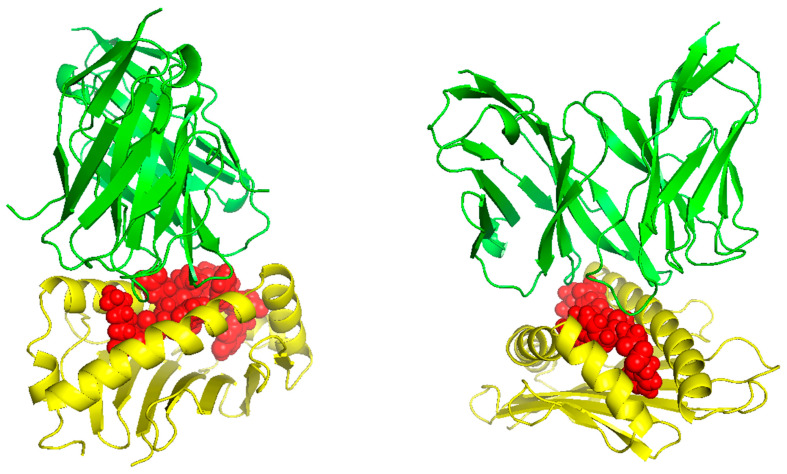
The peptides KSFEDIHHY and KTYQGSYGF bound to HLA-B*58:01 and their respective TCR complex.

TCR (T-cell receptor) and pMHC (peptide-major histocompatibility complex) interactions play a fundamental role in immunogenicity, which involves the ability of a peptide to initiate an immune response against tumor cells. TCRs on the surface of T cells recognize antigens that are presented by MHC molecules on the surface of antigen-presenting cells. This recognition is specific to the peptide being presented by the MHC. The correct configuration and interaction of a TCR with a pMHC complex is essential for the T cell to become activated and initiate an immune response. Thus, to explore the binding of our designed peptide vaccines, we initiated TCR-pMHC peptide interaction modeling. We found the PDB files for the HLA alleles HLA-B*58:01 on the RCSB protein data bank https://www.rcsb.org/ (accessed on 23 September 2023). Using MDockPeP https://zougrouptoolkit.missouri.edu/mdockpep/ (accessed on 23 September 2023) and CABS-dock [25,26], we attached a top epitope to the binding grooves of the HLA allele. We created two models of the peptide-MHC binding complex (Figure 4). TCR binding models were created using the same method as Kim et al. [21]. We used TCRModel https://tcrmodel.ibbr.umd.edu/ (accessed on 23 September 2023) to create 3D models of a TCR complex binding to our peptide-MHC complexes. Subsequently, we used PyMOL version 2.5.5 to edit all of the 3D models. In Figure 4, yellow color represents HLA alleles, and red represents epitopes. The 3D models we obtained were KSFEDIHHY, a mutation of the KRAS gene, binding to the MHC Class I molecule HLA-B*58:01 as well as KTYQGSYGF, a mutation of the TP53 gene, binding to the MHC Class I molecule HLA-B*58:01. All pMHC-TCR 3D molecular model structures generated in this study can be found in Figure 5 and Supplementary Files S6–S9.

## 6. Discussion

We developed a personalized peptide-based vaccine for two patients with pancreatic ductal adenocarcinoma (PDAC). This process began with RNA sequencing (RNAseq) analysis, which enabled the identification of specific genetic mutations driving the development of PDAC in the patients. Based on this analysis, we developed a personalized cancer vaccine using our previously published peptide vaccine development strategy. Our approach involved targeting 100 epitopes that were prevalent in the PDAC patient and identified as viable candidates for peptide vaccine design. By focusing on the specific gene targets present in each patient, we intended to improve the specificity of the vaccine, ensuring that it effectively targeted the unique genetic alterations present in the patient’s tumor. This method not only enhances the potential efficacy of the vaccine by adapting it to the individual’s genetic landscape but also minimizes potential off-target effects, thus optimizing the therapeutic outcome.

The final filtered epitopes are predicted to be immunogenic and antigenic, have a high or normal binding affinity, and are non-toxic and non-allergenic. The binding affinity restriction used in this study differs from other previous in silico vaccine design methodologies using the same NetMHC tool. Our previous methods of peptide vaccine design used quantitative filters on the percentile rank of the binding affinity value. However, the percentile rank compares the epitopes to a test set of data in IEDB and, therefore, is not an accurate nor absolute assessment of binding affinity necessary for this study. Using the IC_50_ value instead is an absolute measure of the binding affinity of the epitopes. We are also able to specify the strength of the binding affinity based on the IC_50_ value, which provides more qualitative measures for comparison when transitioning to murine studies. By tailoring the vaccine to each patient’s specific genetic makeup, we expect to enhance its effectiveness and improve clinical outcomes. This approach represents a significant step forward in the field of immunotherapy for PDAC, offering a more targeted and personalized treatment option that has the potential to transform the management of this challenging disease.

The top epitopes selected using our novel methodology are widely recognized in the literature as common drivers and tumor-suppressor genes in PDAC [9,27,28]. Additionally, these specific epitopes have been identified in trials involving the sequencing of human tumor samples [29,30]. The consistent presence of our top epitopes in both our reference study and other clinical trials of PDAC patients serves as strong validation of our personalized cancer vaccine design methodology. Using RNA sequencing analysis by Partek Flow, along with our peptide cancer vaccine design processes, we created a peptide vaccine derived from the individual’s tumor tissue genetic data. This integrative approach not only emphasizes the relevance of our vaccine targets but also enhances the precision medicine framework by adapting the therapeutic strategy to the genetic individualities of each patient’s tumor. This could potentially lead to improved clinical outcomes by specifically targeting the molecular abnormalities driving the cancer.

Previous studies on the development of peptide vaccines have primarily concentrated on creating generalized vaccines that could be used for a large and broad population [15,31,32,33]. These generalized vaccines target a limited set of gene mutations to increase sensitivity but often at the expense of specificity. The development of effective global peptide vaccines poses additional challenges. The vast global diversity of HLA alleles complicates the creation of a peptide vaccine that can effectively target a comprehensive population [31]. Each individual’s HLA type influences how well their immune system can recognize and respond to the peptides presented by the vaccine, making it difficult to design a universally effective vaccine. The development of personalized peptide vaccines has historically been limited by the cost and time to produce the peptides [32]. However, implementing a novel design method described in this study offers a unique and innovative solution to quickly design neoantigen personalized peptide-based vaccines. Recently, with the advent of advanced sequencing technology, neoantigen peptide vaccines are becoming a more viable solution for patients [34]. However, the design process has been complicated by a multitude of software required to design a personalized vaccine. Our methodology using Partek Flow provides a simple and streamlined RNAseq analysis procedure to obtain the list of neoantigens. Our program, SCGeneID, is useful for identifying and confirming proper alignment and identification of genes from the RNAseq analysis process. Overall, our methodology employs only two tools throughout the entire design process, significantly simplifying the development of personalized cancer vaccines. This streamlined approach not only reduces the complexity and duration of vaccine design but also enhances the precision with which these vaccines can be personalized to individual genetic profiles.

## 7. Limitations

While this study presents a promising personalized cancer vaccine strategy targeting neoantigens in pancreatic ductal adenocarcinoma (PDAC) patients, there are several limitations that should be acknowledged. Firstly, the pilot trial size of two patients is relatively small, which could limit the generalizability of the methodology. A larger sample size would provide more robust data and a better account for variations and accommodate the heterogeneity inherent in the genetic landscape of PDAC more effectively. The purpose of this paper was to demonstrate a successful method for designing personalized peptide cancer vaccines. However, in future outcome-oriented studies, the use of a larger sample size would be favorable. While the absence of experimental confirmation may appear as a limitation, the significance of this innovative methodological framework for personalized cancer vaccines, being the first of its kind, corroborates the importance of this work. This framework enables the efficient prioritization of the most promising personalized vaccine candidates, thus accelerating the vaccine design process, enhancing the probability of success in subsequent preclinical and clinical evaluations, and also helping to optimize resources by focusing on the candidates for further preclinical studies. Peptide vaccines have their weaknesses in functionality as well. If a patient’s cancer significantly downregulates MHC, the probability of a peptide binding to an MHC receptor significantly decreases.

## 8. Future Directions

We have developed an automation of the peptide vaccine design process using web scraping and API tools [24,25]. Implementation of such software would further simplify the personalized cancer vaccine process. Additionally, the use of these programs would allow for the prediction of MHC class II epitopes as well. Furthermore, moving the RNAseq analysis process from a cloud-based solution using Partek Flow to a hardware process using Python or R would allow for complete automation of the personalized vaccine design process. Given such a scaled program and processes, the only limitation to the vaccine design process would be the time to sequence a patient’s tumor tissue.

## 9. Conclusions

We developed a personalized cancer vaccine targeting specific gene mutations prevalent among PDAC patients by implementing our novel personalized vaccine design workflow. This study addresses the limitations of generalized vaccines specifically for pancreatic ductal adenocarcinoma (PDAC). By analyzing the genetic alterations driving PDAC in a patient’s tumor tissue, we identified 100 gene mutations as targets for our personalized vaccine strategy. The gene targets were identified and validated using our SCGeneID program, which used the chromosome number and nucleotide position data. By integrating SCGeneID into our workflow, we not only enhanced the precision of our gene annotations but also significantly improved the efficiency of our data analysis process. This development represents a significant step forward in the application of computational tools in personalized vaccine design, providing a robust method for accurate gene identification and the validity of complex genomic analyses.

The top 50 epitopes consisted of only high-affinity binding epitopes, indicating the potential efficacy of the vaccine. The use of IC_50_ values as an absolute measure of binding affinity provided more accurate and quantitative comparisons. To visualize the interactions between epitopes and HLA alleles, 3D models of TCR-peptide-MHC complexes were created. The personalized cancer vaccine developed in this study may hold great promise for PDAC patients. By targeting the unique genetic alterations in each patient’s tumor, this approach offers a more specific and personalized treatment option. Further research is warranted to simplify the variant identification and epitope ranking process.

## Figures and Tables

**Table 1:** Restriction criteria to quantitatively filter and qualitatively assess each epitope.

Parameter		Restriction
Binding affinity (*b*)	Strong binder	0 nM ≤ *b* ≤ 50 nM
	Normal binder	50 nM < *b* ≤ 500 nM
	Weak binder	500 nM < *b* ≤ 5000 nM
Immunogenicity (*i*)	*i* ≥ 0	
Antigenicity (*a*)	*a* ≥ 0.4	
Toxicity	Non-toxic	
Allergenicity	Non-allergenic	

**Table 2:** Top 50 epitopes along with their strong-binding associated HLA allele.

Gene	Mutation	Epitope	HLA Alleles
GNAS	R201C	AMSNLVPPV	HLA-A*02:01
SMAD4	Y353C	QSIKETPCW	HLA-B*58:01
TP53	R248Q	CTYSPALNK	HLA-A*03:01
KRAS	G12D	KSFEDIHHY	HLA-B*58:01
SMAD4	Y353C	MPIADPQPL	HLA-B*39:01
SMAD4	Y353C	CLSDHAVFV	HLA-A*02:01
SMAD4	Y353C	KIYPSAYIK	HLA-A*03:01
TP53	R248Q	LEDSSGNLL	HLA-B*40:01
KRAS	G12D	LARSYGIPF	HLA-B*15:01
TP53	R248Q	APAAPTPAA	HLA-B*07:02
SMAD4	Y353C	LLDEVLHTM	HLA-A*02:01
TP53	R248Q	KTYQGSYGF	HLA-B*58:01
SMAD4	Y353C	APAISLSAA	HLA-B*07:02
SMAD4	Y353C	LQSNAPSSM	HLA-B*15:01
TP53	R248Q	LLGRNSFEV	HLA-A*02:01
KRAS	G12D	KSALTIQLI	HLA-B*58:01
SMAD4	Y353C	KETPCWIEI	HLA-B*40:01
GNAS	R201C	NQFRVDYIL	HLA-B*39:01
TP53	R248Q	LQIRGRERF	HLA-B*15:01
SMAD4	Y353C	LPHHQNGHL	HLA-B*07:02
SMAD4	Y353C	LQVAGRKGF	HLA-B*15:01
SMAD4	Y353C	CILRMSFVK	HLA-A*03:01
KRAS	G12D	CLLDILDTA	HLA-A*02:01
SMAD4	Y353C	LRRLCILRM	HLA-B*27:05
GNAS	R201C	LIDCAQYFL	HLA-A*02:01

**Table 3:** Population coverage of the personalized PDAC vaccine for regional subgroups.

Population/Area	Coverage	Average Hit	pc90
Central Africa	39.22	1.84	0.16
Central America	1.4	0.06	0.41
East Africa	41.73	2.16	0.17
East Asia	55.26	2.8	0.22
Europe	81.05	4.98	0.53
North Africa	43.55	2.29	0.18
North America	70.36	4.09	0.34
Northeast Asia	47.97	2.21	0.19
Oceania	38.93	1.59	0.16
South Africa	23.99	0.93	0.13
South America	36.87	1.86	0.16
South Asia	37.28	1.66	0.16
Southeast Asia	55.59	2.34	0.23
Southwest Asia	43.73	2.33	0.18
West Africa	42.65	2.14	0.17
West Indies	63.52	3.47	0.27
Average	45.19	2.3	0.23
Standard deviation	17.8	1.13	0.11

## Data Availability

All the data supporting reported results can be found in the Supplementary Materials.

## Supplementary Materials

The following supporting information can be downloaded at https://www.mdpi.com/article/10.3390/diseases12070149/s1, File S1: SCGeneID Python program; File S2: Top 50 Epitopes for Patient 1; File S3: Top 100 Epitopes for Patient 1; File S4: Top 50 Epitopes for Patient 2; File S5: Top 100 Epitopes for Patient 2; File S6: 3-dimensional structure of pMHC complex in Figure 4a; File S7: 3-dimensional structure of pMHC complex in Figure 4b; File S8: 3-dimensional structure of pMHC-TCR complex in Figure 5a; File S9: 3-dimensional structure of pMHC-TCR complex in Figure 5b.
